# Three-Dimensional Printing of Recycled Polypropylene and Activated Carbon Coatings for Harmful Gas Adsorption and Antibacterial Properties

**DOI:** 10.3390/polym15051173

**Published:** 2023-02-26

**Authors:** Jung Bin Park, Seok Hwan An, Jae Woong Jung, Jea Uk Lee

**Affiliations:** Department of Advanced Materials Engineering for Information and Electronics, Integrated Education Institute for Frontier Science and Technology (BK21 Four), Kyung Hee University, 1732 Deogyeong-daero, Giheung-gu, Yongin-si 17104, Gyeonggi-do, Republic of Korea

**Keywords:** 3D printing, recycled polymer, activated carbon, carbon coating, gas adsorption, antibacterial

## Abstract

In recent years, the utilization of three-dimensional (3D) printing has been expanding due to advances in technology and economic efficiency. One of the 3D printing technologies is fused deposition modeling, which can be used to create different kinds of products or prototypes from various polymer filaments. In this study, the activated carbon (AC) coating was introduced to the 3D outputs printed using recycled polymer materials to impart multi-functions such as adsorption of harmful gas and antimicrobial activities. A filament of uniform diameter (1.75 μm) and a filter template in the form of a 3D fabric shape were prepared through the extrusion and 3D printing processes, respectively, of the recycled polymer. In the next process, the 3D filter was developed by coating the nanoporous AC, produced from the pyrolysis fuel oil and waste PET, on the 3D filter template through direct coating. The 3D filters coated with the nanoporous activated carbon showed the enhanced adsorption capacity of 1038.74 mg of SO_2_ gas and the antibacterial properties of 49% removal of *E. coli* bacteria. As a model system, a functional gas mask that has harmful gas adsorption abilities and antibacterial properties has been produced by a 3D printing process.

## 1. Introduction

The manufacturing of plastic goods has greatly increased on a global scale. The Plastic Europe Market Research Group estimates that 359 million tons of plastic were produced worldwide in 2019, with Asia accounting for 51% of the total and Europe for 17%. The plastic manufacturing industries have used 1.3 billion barrels of oil annually or 4% of the world’s total oil production [1]. The polymers used in plastic manufacturing are chemically inert and can last a very long time as their unaltered forms in the air and the ground [2]. Therefore, global environmental pollution due to the enormous amount of polymer waste is currently at a very serious level and must be overcome through the recycling of polymers. Statistically, more than 90% of the polymer can be recycled; however, only a small portion of polymer garbage is recycled and up to 80% is buried underground.

The recent development of three-dimensional (3D) printing technology for thermoplastic polymer filaments has opened a new route for recycling waste polymers. The process of layer-by-layer fabrication of 3D structures directly from computer-aided design blueprints is known as 3D printing [3]. In recent years, 3D printing has seen many significant expansions since Charles Hull [4] successfully marketed 3D printing in 1980. Thermoplastic filaments, such as polylactic acid (PLA) and acrylonitrile butadiene styrene (ABS), are the most commonly used sources in 3D printing, known as the fused deposition modeling (FDM) method [5]. Polycaprolactone, polycarbonate, polystyrene (PS), polyetherimide, polyether ether ketone, polypropylene (PP), and low-density and high-density polyethylene (LDPE and HDPE) are among the remaining material categories in polymer 3D printing technology [6]. The realm of the polymer 3D printing technology has evolved into one that is creative and adaptive to new businesses such as jewelry collectibles [7], 3D printed artificial heart pumps [8] and corneas [9], and rocket engines [10].

Recently, the most widely used polymer filaments for 3D printing have been commercialized from recycled polymers, such as PLA (Maker Geeks, Springfiled, IL, USA) and ABS (Kickfly^,^ Boston, MA, USA). In addition, some researchers have described the utilization of recycled polymers (LDPE and polyethylene terephthalate (PET)) as the feedstocks for 3D printing [11,12]. Zander et al. have reported the 3D printing of recycled blends from consumer-grade polymers [13]. They used the recycled PP/PET and PP/PS blends, compounded with or without a compatibilizer, and evaluated the mechanical and thermal properties. However, the introduction of functional materials onto the 3D outputs, printed with filaments based on recycled polymers, has not been reported.

**Figure 1:**
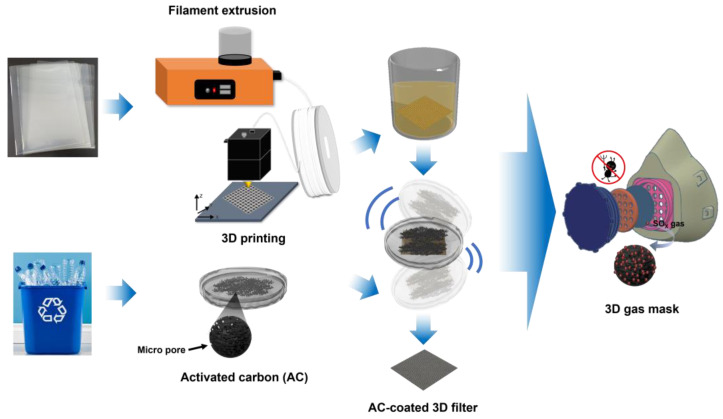
Preparation process of a 3D gas mask from recycled polymers.

Here, we report the studies imparting new properties to the 3D printed recycled polymers by introducing nanoporous activated carbon particles (Figure 1). To prepare the recycled polymer filament, the PP chips from laboratory waste were melt extruded using a single-screw extruder. This recycled PP filament was 3D printed to manufacture two types of 3D filter templates of a simple plate shape and fabric shape. In addition, the activated carbon synthesized from the waste PET was introduced onto the 3D printed filter template through the direct coating process. To confirm the applicability of the fabricated 3D filter to the gas mask, the toxic SO_2_ gas adsorption abilities and antibacterial properties were evaluated.

## 2. Experimental section

### 2.1. Materials

The recycled polymers were obtained from laboratory waste. A commercial activated carbon (cAC) was supplied from GEO Nation Co., Ltd. (Incheon, Republic of Korea), while the gelatin glue was purchased from Kissho company (Aichi, Japan). Anhydrous ethyl alcohol (C_2_H_5_OH, 99.9%) was purchased from Samchun Chemical Co., Ltd. (Seoul, Republic of Korea). ABS filament for 3D printing was purchased from Cubicon Inc. (Seoul, Republic of Korea). Luria-Bertani (LB) broth, a microbial growth medium used for the cultivation of *E. coli*, was purchased from Sigma-Aldrich Inc. (St. Louis, MO, USA).

### 2.2. Synthesis of Activated Carbon Based on Recycled Polymer

Activated carbon synthesized by adding recycled polymer (rAC) was prepared using a previously reported method [14]. Briefly, pyrolysis fuel oil (LG Chem., Seoul, Republic of Korea) and waste PET were prepared as precursors for the manufacturing pitch. The pitch was synthesized at 420 °C for 3 h, which is the temperature and time at which PET can fully degrade. The PET addition amount was 10 wt%. Then, 20 g of the pitch and 80 g of sodium hydroxide (NaOH, 98%, Alfa Aesar, Ward Hill, MA, USA) were activated and carbonized at 800 °C for 1 h in an inert atmosphere.

### 2.3. Filament Extrusion with Recycled Polymer

**Figure 2:**
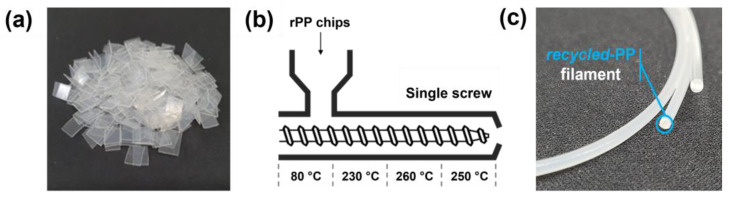
(**a**) Photograph of rPP chips. (**b**) Single screw extruder with heating temperature profiles. (**c**) Photograph of rPP filament.

To fabricate the filament, the feedstock was obtained from an opaque polypropylene L-shaped file (rPP). The polymers were obtained from laboratory wastes and cut to a size of 1 × 1 cm^2^ (Figure 2a). The rPP chips were cleaned by rinsing with water and ethanol and then dried in a vacuum oven at 80 °C for 24 h to avoid moisture-induced degradation. A recycled filament was prepared via an extrusion process conducted using a single-screw extruder (3D Factory, Inc., Paju, Republic of Korea, φ = 20 mm, L/D = 27) (Figure 2b). The temperature profiles of the extruder from zones 1 to 4 were set at 80 °C, 230 °C, 260 °C, and 250 °C, respectively. The screw rotation speed and collection rate were regulated to obtain a final diameter of the extruded filament of 1.75 ± 0.10 mm, with an output of 110 rpm. The extruded strands (average diameter = 1.75 mm) were cooled in a water bath and then used for the 3D printing process (Figure 2c).

### 2.4. Three-Dimensional Printing with Recycled Polymer Filament

**Figure 3:**
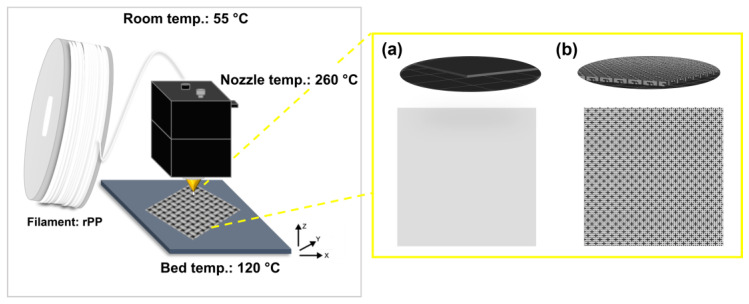
Scheme of 3D printing procedure for 3D filter template; (**a**) plate shape and (**b**) fabric shape.

Before 3D printing, the recycled polymer filament was dried under a vacuum overnight to remove any excess moisture. The dog-bone specimen for tensile tests (ASTM D638) and 3D filter templates (0.05 mm × 50 mm × 50 mm) were printed by feeding the recycled polymer filaments using a commercial desktop FDM printer (Single Plus, Cubicon, Inc., Seongnam-si, Republic of Korea) (Figure 3). At first, the 3D STL files were edited using Cubicreator 4 (Cubicon, Inc., Seongnam-si, Republic of Korea) and the output design was built up along three orientations, i.e., horizontal, vertical, and perpendicular, using the software Fusion 360 (Autodest, Inc., San Rafael, CA, USA). During the 3D printing processes for all samples, the nozzle temperature, bed temperature, printing speed, and layer height were set at 260 °C, 120 °C, 100 mm/s, and 0.15 mm/layer, respectively.

### 2.5. Coating with AC on 3D Printed Filter Template

**Figure 4:**
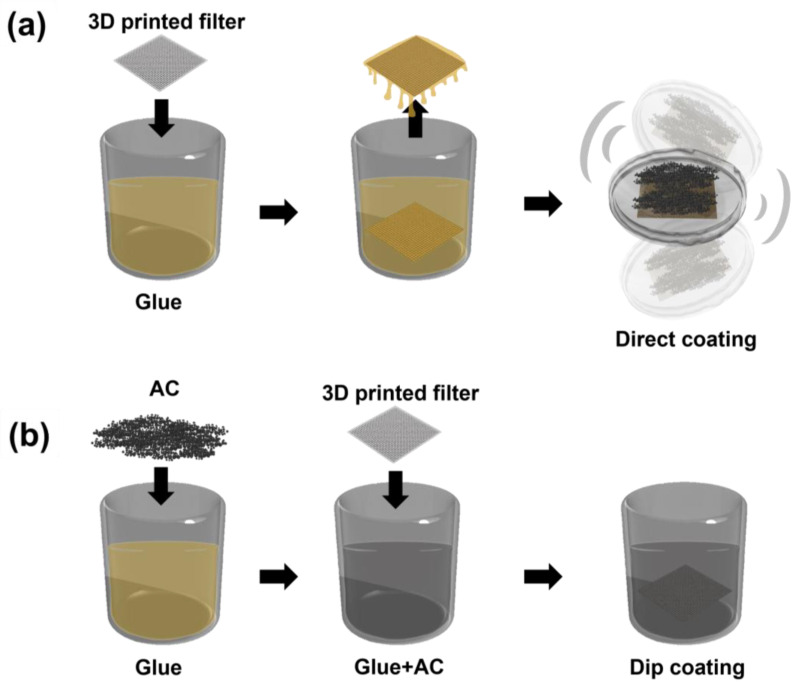
Scheme of AC coating onto 3D printed filter template; (**a**) dip coating (**b**) direct coating.

The 3D printed filter template was rinsed with anhydrous ethyl alcohol, dried at room temperature, and placed in a beaker containing 10 mL of gelatin glue (Figure 4a). Before the gelatin glue on the surface was cured, the 3D printed filter template was directly placed in a petri dish containing 300 mg of AC. The petri dish was manually shaken for 1 min and the AC-coated filter template was retrieved from the petri dish using tweezers, after which it was dried in a vacuum oven at 25 °C for 24 h. After drying, the AC-coated filter template was rinsed again with anhydrous ethyl alcohol. 

For comparison, the dip coating process was also carried out (Figure 4b). After the 3D printed filter template was rinsed with anhydride ethyl alcohol and dried at room temperature, it was directly placed in a beaker containing 300 mg of AC dispersed in 200 mL of gelatin glue and then retrieved from the petri dish using tweezers. It was dried in a vacuum oven at 25 °C for 24 h and then rinsed again with anhydrous ethyl alcohol.

### 2.6. Characterization

Functional groups of the polymers were investigated by Fourier transform infrared spectroscopy (FT-IR, Nicolet 6700, Thermos Fisher Scientific, Inc., Walthanm, MA, USA) in the range of 500–4000 cm^−1^. The thermal properties of the polymers were examined using a differential scanning calorimeter (DSC, Thermo Plus DSC 8230, Rigaku Corporation). Heating was conducted at a heating rate of 10 °C per minute to 275 °C under a nitrogen atmosphere. The molecular weights of the polymers were measured by gel permeation chromatography (GPC, Agilent 1260LC system, Santa Clara, CA, USA) using THF as an eluent. The molecular weights were reported relative to polystyrene standards. The tensile strength of the polymer samples was measured by a tensile test based on ASTSM D638V using a universal tensile machine (5882, Instron, Norwood, MA, USA) with a load cell of 100 kN. Five specimens of each sample were prepared by 3D printing a standard dog-bone shape. The mechanical parameters were the average values from five specimens of each 3D printed output. The pore structure of the AC samples was investigated via nitrogen gas adsorption analysis at 87.34 K using ASAP2020 (Micromeritics, Norcross, GA, USA). The specific surface area of the samples was obtained through the Brunauer–Emmett–Teller (BET) theory. The micropore size distribution of the samples was determined using the Horvath-Kawazoe equation. The microstructures of the AC samples and 3D printed filters were investigated using a scanning electron microscope (SEM) (COXEM, CX-200TA, Daejeon, Republic of Korea). Thermogravimetric analysis (TGA, Thermo plus EVO2 TG 8120 series, Thermos Fisher Scientific, Inc., Walthanm, MA, USA) of the AC-coated 3D filters was performed by heating approximately 3 mg samples at a heating rate of 5 °C/min from room temperature to 900 °C in a nitrogen atmosphere. To measure the harmful gas adsorption abilities, 0.1 g of the AC-coated 3D filter was placed in a vertical stainless-steel reactor (internal diameter of 5 mm). The thermocouple was located 1.0 mm above the 3D sample to measure the internal bed temperature. The total flow was kept constant at 1500 cc/min, the SO_2_ gas concentration was approximately 40 ppm, and the analyzer continuously monitored the weight gain. The outlet SO_2_ gas concentrations were continuously measured by a Teledyne Model 7600 Analyzer. The outlet gas composition was recorded every 1 s. The antibacterial activity of the AC-coated 3D filter against the Gram-negative bacterium *Escherichia coli* (*E. coli)* was investigated using ASTM e2149. The bacterial inhibition was evaluated by counting the number of viable microorganisms present after the 3D samples were placed in a bacterial suspension for 0, 30, 60, and 120 min. The inoculation concentration was approximately 1 × 10^5^ CFU/mL.

## 3. Results and Discussion

### 3.1. Characterization of Recycled Polymer

**Figure 5:**
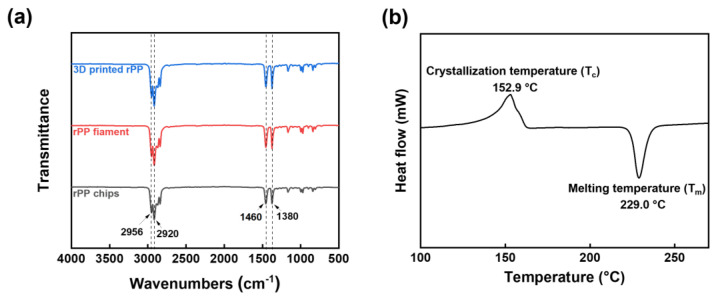
(**a**) FT-IR spectra of rPP chips, rPP filament, and 3D printed rPP. (**b**) DSC spectrum of rPP chips.

The chemical structures of the recycled PPs were identified by FT-IR, as shown in Figure 5a. A comparison of the FT-IR spectra of the rPP chip, rPP filament prepared by the single-screw extrusion process, and 3D printed rPP reveals that the peaks of the three spectra are nearly identical. The main characteristic peaks of the rPPs are located at 2956, 2920, 1460, and 1380 cm^−1^, corresponding to the stretching vibrations of the alkyl main and side chains (CH_3_, CH_2_, and C-H segments), respectively, which are the same as the typical FT-IR spectrum of a virgin PP [15]. Therefore, this confirms that the recycled chips are polypropylene-derived polymers. Furthermore, thermo-oxidative changes of the recycled polymers during some heating processes (single-screw extrusion and 3D printing processes) can be monitored by FT-IR spectroscopy. Typically, the carbonyl peak in the range of 1650–1850 cm^−1^ appears when the polymeric materials are oxidized [16]. Since no clear carbonyl peak was found in the FT-IR spectra of the rPP filament and the 3D printed rPP, it was confirmed that the samples were not chemically altered by the heating processes.

Figure 5b shows the DSC curve of the rPP chips, where the crystallization and melting temperatures are 152.9 °C and 229.0 °C, respectively [17]. In addition, there was no reaction up to the DSC analysis temperature of 275 °C. Since both the extrusion and 3D printing temperatures (260 °C) were determined through the DSC analysis, no thermal oxidation reaction occurred.

**Figure 6:**
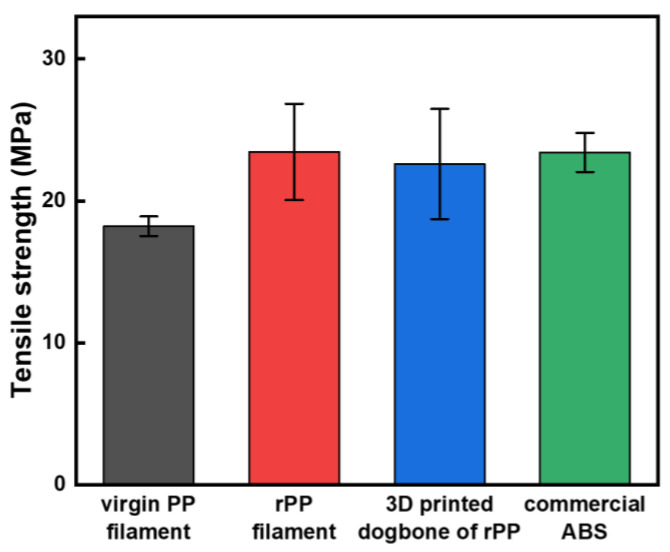
Tensile strengths of virgin PP filament, rPP filament, 3D printed dog-bone of rPP, and commercial ABS.

To further analyze the effect of the heating processes on the mechanical properties of the recycled polymers, a standard tensile test of the filament and 3D printed dog-bone was performed (Figure 6). As shown in Figure S1, the printing orientation was set up in the parallel direction of the tensile test to yield the maximum strength. The tensile strengths of the filaments manufactured through the extrusion process of virgin PP and rPP were 18.21 MPa and 23.45 MPa, respectively. In addition, the strength of the 3D printed rPP showed only a small change of 3.62% compared to that of the rPP filament. From these results, it was confirmed that there was no significant effect on the mechanical properties of rPPs through the heating processes, such as single-screw extrusion and 3D printing. Recycled plastics typically exhibit lower mechanical strength and thermal resistance than those of the virgin materials, since they are exposed to a lot of heat and shear stress in the reprocessing procedures [18,19,20,21]. For this reason, virgin polymers are sometimes added in the plastic recycling processes to meet the acceptable mechanical properties [22]. However, the yield strength of the rPP filament used in this study was higher than that of the virgin PP filament, presumably due to the difference in the molecular weight (number average molecular weight of 2.10 × 10^3^ for virgin PP filament and 4.54 × 10^3^ for rPP filament, Figure S2), and the deterioration in strength was minimized due to the elaborately designed heat treatment processes. Furthermore, there was no significant difference in the yield strengths compared to that of the 3D printed dog-bone of ABS (23.42 MPa), one of the most widely used polymers in FDM 3D printing. Therefore, it is expected that the 3D outputs made of the rPP filaments can be applied to various uses in everyday life, such as filters and masks.

### 3.2. Characterization of Activated Carbon

**Figure 7:**
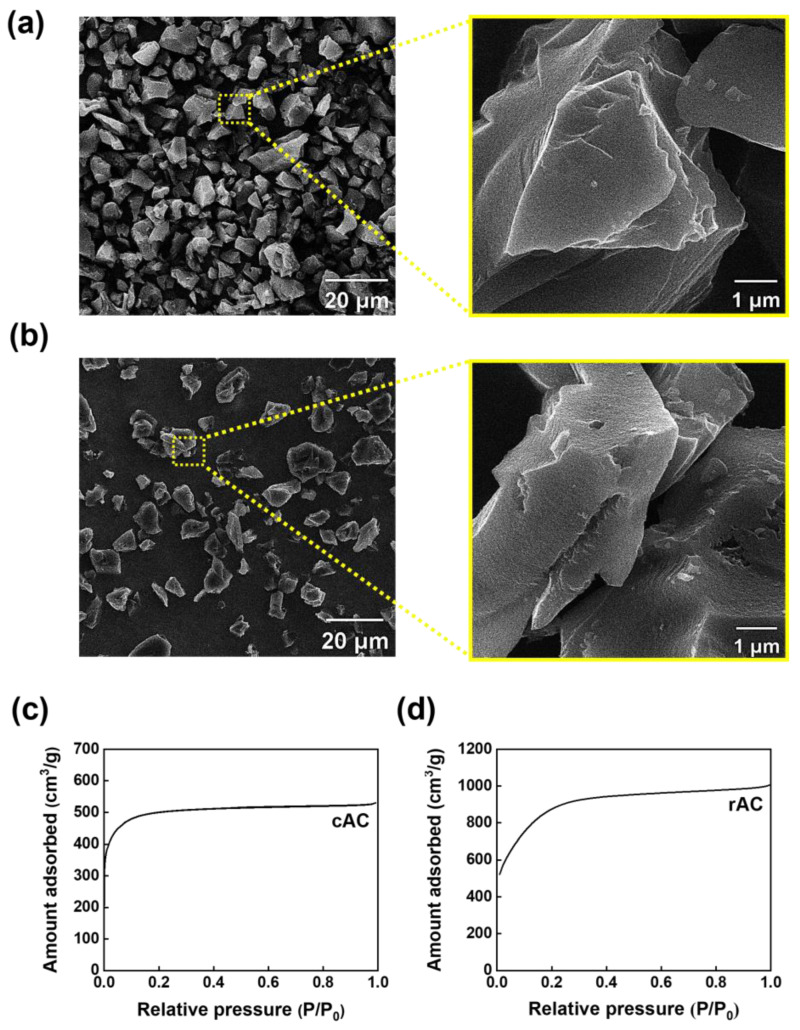
SEM images of (**a**) cAC and (**b**) rAC. Nitrogen adsorption isotherms of (**c**) cAC and (**d**) rAC.

The morphological characteristics of the cAC and rAC, which were synthesized from the petroleum pitch and wasted PET, were performed using SEM (Figure 7). The cAC has a flake structure with a particle size of 10~15 μm (Figure 7a). Although the experimentally prepared rAC shows a very similar structure and particle size (Figure 7b), the pore size studied by nitrogen adsorption analyses is very different. Table 1 lists the specific surface area, micropore volume, total pore volume, and micropore fraction of the ACs. The rAC had a high micropore faction of more than 90% and showed a much higher specific surface area (3298 m^2^ g^−1^) than that of cAC (1849 m^2^ g^−1^). From these observations and analyses, it was confirmed that the rAC has a well-developed micropore structure that is advantageous for adsorbing harmful gases, such as SO_x_ and NO_x_ [23,24].

### 3.3. Preparation and Characterization of AC-Coated 3D Filter

**Figure 8:**
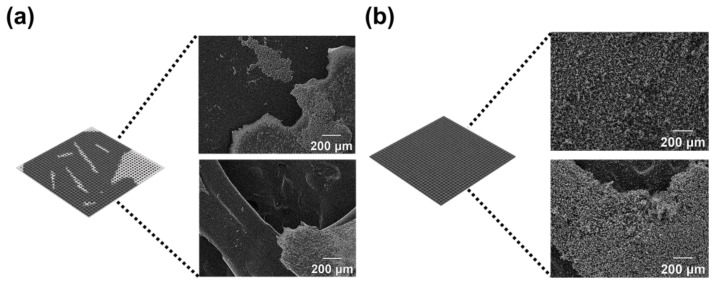
SEM images of AC-coated 3D filter templates using (**a**) dip coating and (**b**) direct coating methods.

Figure 8 shows the SEM images of the coating and adhesion of the AC to the 3D printed filter templates in two different methods, dip coating and direct coating. As a result of placing the 3D printed filter template directly to the gelatin glue in which the AC was dispersed (dip coating method), the AC was not uniformly coated on the filter template because of the three-dimensional and complex structure of the 3D filter template (Figure 8a). To solve this problem, AC was applied directly to the 3D filter template coated with semi-cured gelatin glue through the direct coating process. As shown in Figure 8b, the new coating process allowed for the very uniform coating of the AC on the 3D filter without gaps. Therefore, all of the following studies were conducted with direct coating method; the cAC and the synthesized rAC were introduced to the 3D printed plate shape and fabric shape filter templates, respectively, using the direct coating process.

**Figure 9:**
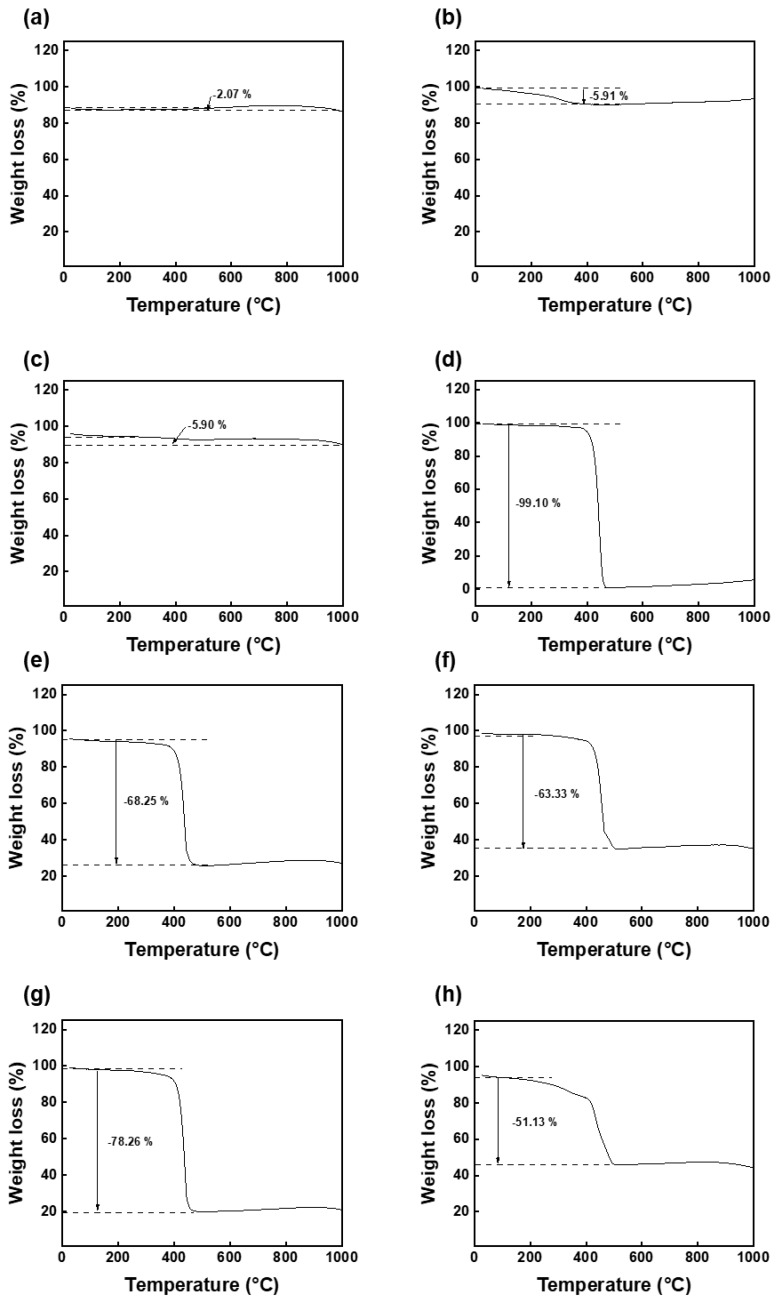
TGA results of (**a**) cAC, (**b**) rAC, (**c**) gelatin glue, (**d**) rPP, (**e**) cAC-coated plate shape filter and (**f**) fabric shape filter, (**g**) rAC-coated plate shape filter and (**h**) fabric shape filter.

The content of the ACs introduced on the 3D filter templates, according to the type of the prepared ACs (cAC and synthesized rAC) and the shape of the filter templates (plate shape and fabric shape) was quantitatively measured using TGA analyses. Both ACs and gelatin glue showed a slight weight loss (2.07% for cAC, 5.91% for rAC, 5.90% for gelatin glue) due to the presence of a small amount of water in the nitrogen atmosphere (Figure 9a–c). On the other hand, the raw material of the filter template (rPP) was completely decomposed around 450 °C, showing a weight loss of 99.10% (Figure 9d). Therefore, under the following assumptions, the mass fraction of the coated carbon on the 3D filter templates could be calculated from the weight loss of the AC-coated 3D filters at the same temperature [25,26].

(1) AC does not lose weight when subjected to the heating program under nitrogen. However, the starting point of weight loss should be calculated from the part where the retained water is removed.

(2) The measured weight loss of the AC-coated 3D filters is proportional to the weight fraction of the polymer.

From the above assumptions, the carbon contents of the cAC-coated filters with plate shape and fabric shape were 31.75% and 36.67%, respectively. Approximately 5% more cACs were introduced onto the 3D filter template with the fabric shape, which has a larger contact area than that of the plate shape filter. On the other hand, the carbon content of the rAC-coated filters with plate shape and fabric shape was recorded as 21.74% and 48.87%, respectively. Further studies are needed on the difference in the carbon contents according to the type of ACs. However, in common, it was confirmed that more ACs were introduced onto the fabric shape filter than the plate shape, and the rAC-coated fabric shape filter contained the largest number of functional areas.

### 3.4. Harmful Gas Adsorption Abilities and Antibacterial Properties of AC-Coated 3D Filter

**Figure 10:**
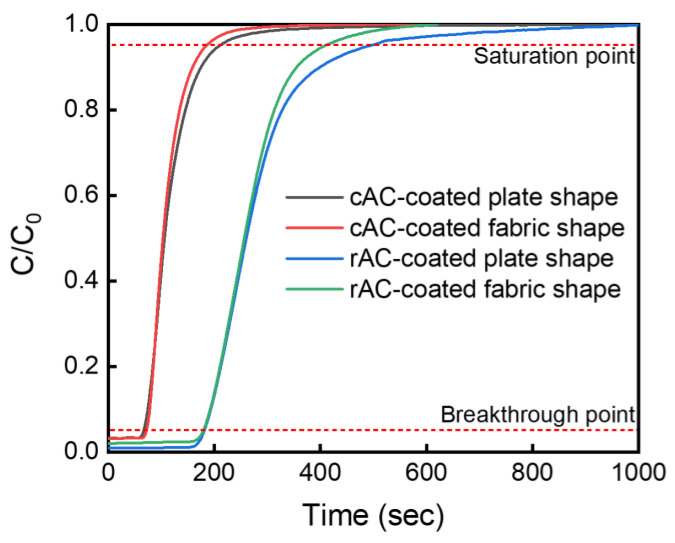
SO_2_ gas absorption behavior curves of AC-coated 3D filters. Dotted lines define the breakthrough and saturation points.

To analyze the SO_2_ gas adsorption abilities of the AC-coated 3D filters, the breakthrough and saturation curves (C_t_/C_0_ vs. time) were drawn, as shown in Figure 10 [27]. From these curves, the breakthrough time, saturation time, percentage of SO_2_ removal, and adsorption capacity of the prepared AC-coated 3D filters were derived (Table 2). In this study, the breakthrough time (t_0.05_) and saturation time (t_0.95_) were defined arbitrarily as the times corresponding to C/C_0_ = 5% and 95%, respectively. As the type of the coated AC was changed from the cAC to the synthesized rAC, both the breakthrough time and saturation time increased significantly. On the other hand, the results according to the type of 3D filter (plate shape and fabric shape) exhibited insignificant differences. In fact, the cAC-coated plate shape filter showed a slightly lower adsorption capacity (572.52 mg) than that of the fabric shape filter (611.88 mg). This is because there is little difference in the carbon content of the cAC-coated filters with plate shape (31.75%) and fabric shape (36.67%), according to the results of TGA analyses. In contrast, when the rAC was used instead of the cAC, the adsorption capacity increased dramatically because of the much higher specific surface area of rAC (3298 m^2^ g^−1^) than that of cAC (1849 m^2^ g^−1^). Finally, the rAC-coated fabric shape filter showed superior adsorption properties (percentage removal of SO_2_ gas of 1.73% and adsorption capacity of 1038.74 mg) to the other filters. From these results, it was confirmed that the carbon content according to the shape of the 3D filter and the specific surface area of the AC used are critical factors for the adsorption of SO_2_ gas.

**Figure 11:**
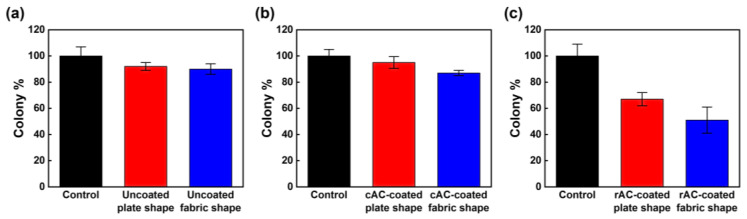
Antibacterial properties of AC-coated 3D filters. (**a**) Uncoated plate shape filter (red) and uncoated fabric shape filter (blue), (**b**) cAC-coated plate shape filter (red) and cAC-coated fabric shape filter (blue), (**c**) rAC-coated plate shape filter (red) and rAC-coated fabric shape filter (blue).

The antimicrobial effects of the AC-coated 3D filters against the Gram-negative bacterium *E. coli* were investigated using ASTM e2149. To check out the antibacterial properties of the rPP used, two uncoated filter templates were tested (Figure S3). As shown in Figure 11a, both polymer filter templates exhibited negligible antibacterial performances with the 8% removal of the colony for the uncoated plate shape filter and 10% removal for the uncoated fabric shape filter. In addition, the cAC-coated filters also did not show any obvious antibacterial properties (13% removal for the cAC-coated plate shape filter and 5% removal for the cAC-coated fabric shape filter) (Figure 11b). On the other hand, the plate shape filter coated with rAC revealed significantly improved antibacterial properties (33% removal) and the fabric shape filter coated with more rAC content killed approximately half of the bacteria (49% removal) (Figure 11c). It has been reported that Gram-negative bacteria such as *E. coli* are affected by high surface areas and ionic functional groups [28]. The rAC used in this study not only has a very large specific surface area but also contains a significant amount of oxygen-containing functional groups due to the introduction of the recycled PET during the pitch synthesis process and the post activation process (Figure S4). Therefore, it was confirmed that the 3D filters containing rAC had significant antibacterial properties.

### 3.5. Application of AC-Coated 3D Filter to 3D Printed Gas Mask

**Figure 12:**
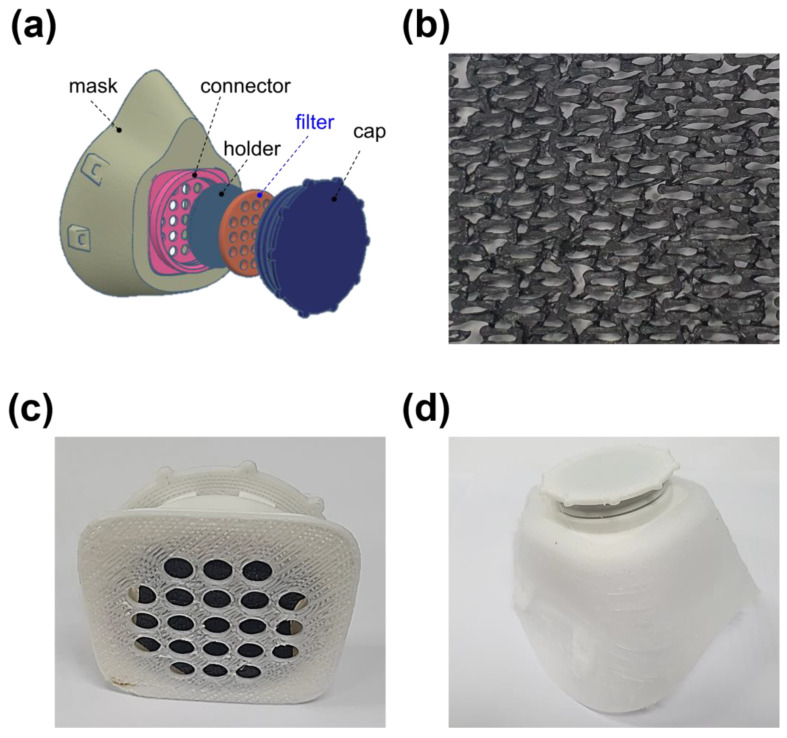
(**a**) Schematic of the gas mask and photo images of (**b**) rAC-coated filter, (**c**) 3D printed cap, and (**d**) 3D printed gas mask.

As a model system, the AC-coated 3D filter was applied to the gas mask, prepared via the FDM 3D printing process. Each part of the gas mask was manufactured by 3D printing with the rPP filament used in this study and assembled to develop the 3D gas mask (Figure 12). The rAC-coated fabric shape filter with excellent SO_2_ adsorption and antibacterial properties was utilized as a filter, a key part of the gas mask. From the model system, it can be expected that the assembly of the 3D printed outputs and the nano-porous activated carbon, prepared from the recycled polymers, is applicable to various applications, such as portable air purifiers and personalized water purification systems [29].

## 4. Conclusions

In this study, AC-coated 3D filters were developed by coating the rAC onto the 3D printed rPP filter template and they were applied to the gas mask which had toxic SO_2_ adsorption and antibacterial properties. Using recycled polymers is a cost-effective method to decrease plastic waste and promote the reuse of these materials. The filament and 3D printed dog-bone, prepared from the recycled PP, exhibited yield strengths of 23.45 MPa and 22.6 MPa, respectively, which are comparable to that of the 3D printed dog-bone of commercial ABS. In the next process, the synthesized AC, based on the waste PET, was coated on the 3D filter templates through direct coating. The rAC-coated fabric shape filter exhibited outstanding adsorption performances (1.73% removal of SO_2_ gas and adsorption capacity of 1038.74 mg), due to the high carbon content (48.87%) and specific surface area of rAC (3298 m^2^ g^−1^). In addition, this filter also showed superior antibacterial properties, with the 49% removal of *E. coli* bacteria compared to other filters. Finally, the 3D filter developed in this study was applied to the gas mask manufactured through 3D printing of rPP-based filaments. Although the developed 3D filter has many points to be addressed for the application as the gas mask filter, through the 3D printing of the waste polymers and the introduction of functional materials, it is believed that various environmental applications such as air and water purification will be possible.

## Figures and Tables

**Table 1:** Summary of argon adsorption–desorption data for AC samples.

	S_BET_ (m^2^/g)	V_mic_ (cm^3^/g)	V_total_ (cm^3^/g)	Micropore Fraction (%)
cAC	1849	0.74	0.76	97.4
rAC	3298	1.42	1.55	91.6

*S*_BET_: BET surface area; V_mic_: micropore volume; V_total_: total pore volume; micropore fraction: micropore volume/total pore volume * 100.

**Table 2:** Summary of SO_2_ gas absorption data for AC-coated 3D filters.

	Breakthrough Time	Saturation Time	Percentage of SO_2_ Removal	Adsorption Capacity
	t_0.05_	t_0.95_	R_SO2_	q_total_
	(min)	(min)	(%)	(mg)
cAC-coated plate shape	67	185	0.95	572.52
cAC-coated fabric shape	73	210	1.02	611.88
rAC-coated plate shape	179	409	1.49	891.77
rAC-coated fabric shape	181	493	1.73	1038.74

## Data Availability

Not applicable.

## Supplementary Materials

The following supporting information can be downloaded at: https://www.mdpi.com/article/10.3390/polym15051173/s1, Figure S1: (a) 3D printing profile, (b) Photo image, and (c) optical microscopy image of 3D printed dogbone. Figure S2: GPC traces of (a) virgin PP filament and (b) rPP filament. Figure S3: Photo images of antibacterial tests of uncoated and AC-coated 3D filters. Figure S4: FT-IR spectra of (a) cAC and (b) rAC [30].
