# Supplementary material for: Three-Dimensional Printing of Recycled Polypropylene and Activated Carbon Coatings for Harmful Gas Adsorption and Antibacterial Properties
Source: Polymers (Basel). 2023 Feb 26;15(5):1173. doi: 10.3390/polym15051173 (PMC10006920; doi:10.3390/polym15051173)
Supplement: Supplementary file 1 [file polymers-15-01173-s001.zip › polymers-2220334-supplementary.pdf]

Supplementary data for

Three-Dimensional Printing of Recycled Polypropylene and Activated Carbon Coatings for Harmful Gas Adsorption and Antibacterial Properties

Jung Bin Park, Seok Hwan An, Jae Woong Jung * and Jea Uk Lee *

Department of Advanced Materials Engineering for Information and Electronics, Integrated Education Institute for Frontier Science and Technology (BK21 Four), Kyung Hee University, 1732 Deogyeong-daero, Giheung-gu, Yongin-si 17104, Gyeonggi-do, Republic of Korea

* Correspondence: wodndwjd@khu.ac.kr (J.W.J.); leeju@khu.ac.kr (J.U.L.); Tel.: +82-31-201-3655 (J.U.L.)

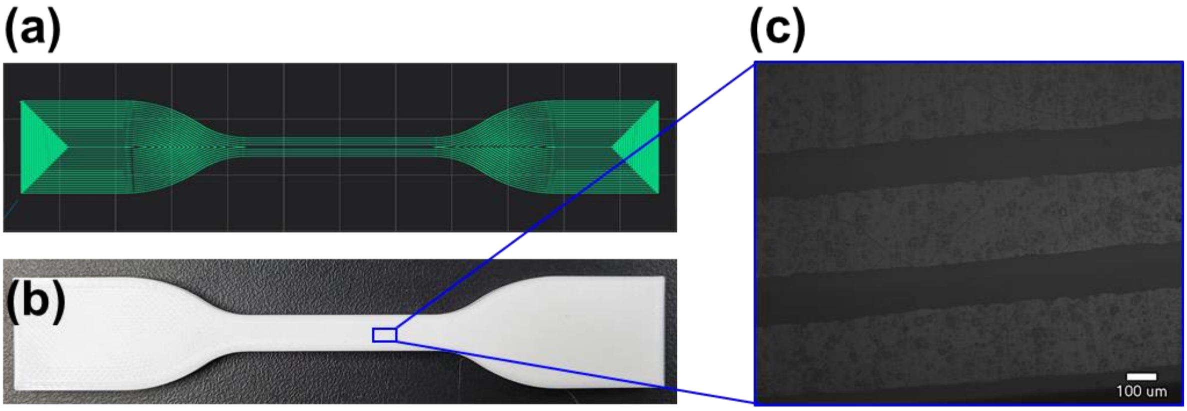

Figure S1. (a) 3D printing profile, (b) Photo image, and (c) optical microscopy image of 3D printed dogbone.

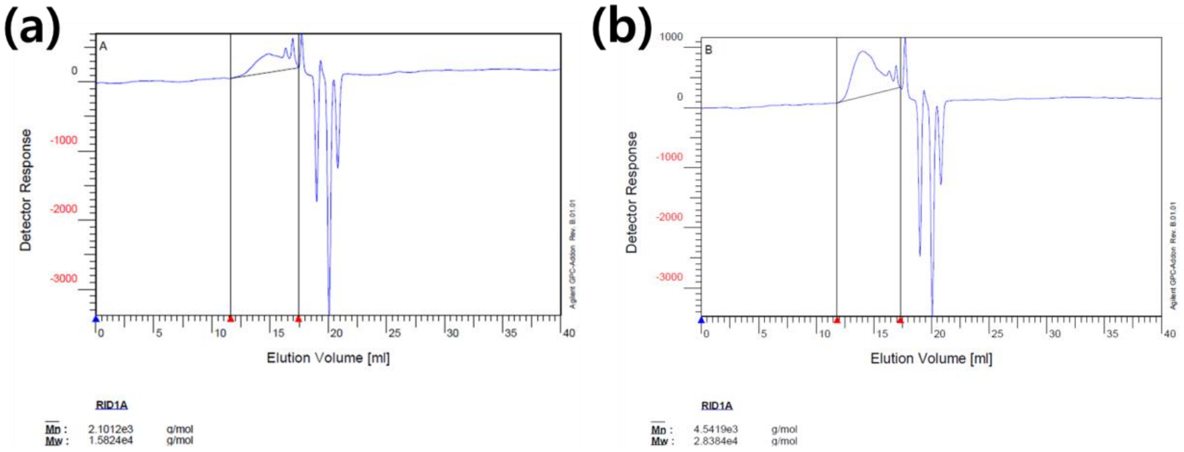

Figure S2. GPC traces of (a) virgin PP filament and (b) rPP filament.

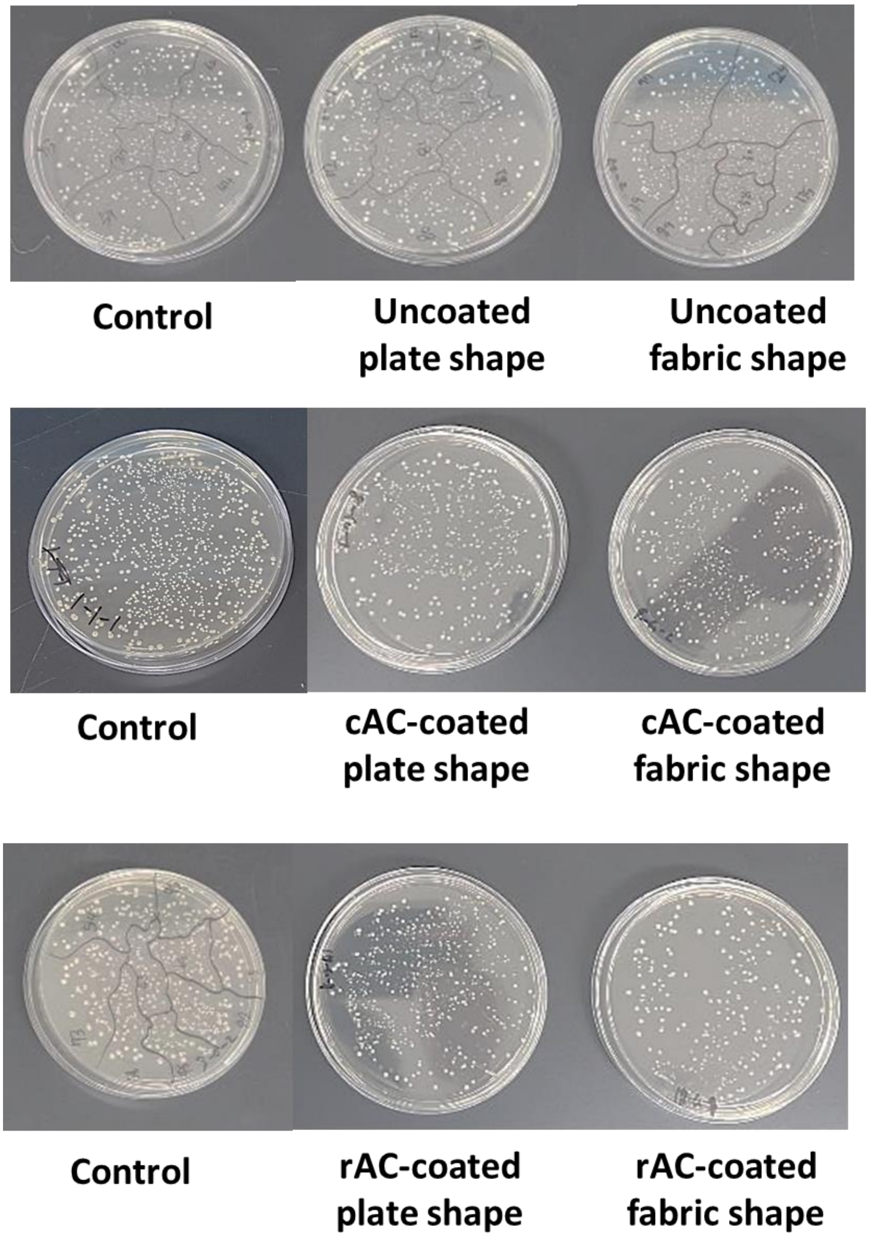

Figure S3. Photo images of antibacterial tests of uncoated and AC-coated 3D filters.

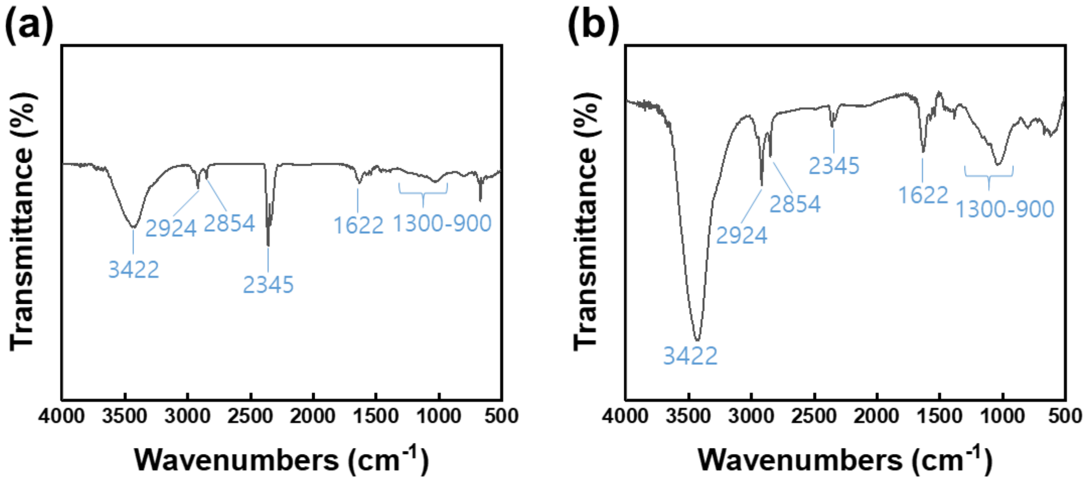

Figure S4. FT-IR spectra of (a) cAC and (b) rAC.

FT-IR analysis was used for identifying surface functional groups of the cAC and rAC. When the spectrum of the rAC is compared with that of the cAC spectrum, it reveals that three characteristic bands corresponding to O−H stretching (3422 cm^{-1}), C=O stretching (1622 cm^{-1}), and C–O stretching vibrations (1300-900 cm^{-1}) of the rAC specimen are much larger than those of the cAC specimen. On the other hand, the C=C peak (2345 cm^{-1}) of the cAC is larger than that of the rAC. Therefore, it can be concluded that the rAC contains a larger amount of oxygen-containing functional groups than the cAC specimen.

References

1. Mojoudi, N.; Mirghaffari, N.; Soleimani, M.; Shariatmadari, H.; Belver, C.; Bedia, J. Phenol adsorption on high microporous activated carbons prepared from oily sludge: equilibrium, kinetic and thermodynamic studies. *Sci. Rep.* **2019**, *9*, 19352. https://doi.org/10.1038/s41598-019-55794-4.
